# Complete Genome Sequence of an *Edwardsiella piscicida*-Like Species Isolated from Diseased Grouper in Israel

**DOI:** 10.1128/genomeA.00829-15

**Published:** 2015-07-23

**Authors:** Stephen R. Reichley, Geoffrey C. Waldbieser, Michal Ucko, Angelo Colorni, Lidiya Dubytska, Ronald L. Thune, Mark L. Lawrence, Matt J. Griffin

**Affiliations:** a College of Veterinary Medicine, Mississippi State University, Mississippi, USA; b USDA-ARS Warmwater Aquaculture Center, Stoneville, Mississippi, USA; c Israel Oceanographic and Limnological Research, National Center for Mariculture, Eilat, Israel; d School of Veterinary Medicine, Louisiana State University, Baton Rouge, Louisiana, USA

## Abstract

The *Edwardsiella piscicida*-like sp. is a Gram-negative facultative anaerobe that causes disease in some fish species. We report here the complete genome sequence of a virulent isolate from a diseased white grouper (*Epinephelus aeneus*) raised on the Red Sea in Israel, which contains a chromosome of 3,934,167 bp and no plasmids.

## GENOME ANNOUNCEMENT

The *Edwardsiella* genus was first recognized in the 1960s (1) and includes a diverse group of enteric, Gram-negative bacteria infecting a wide range of fishes, reptiles, avians, and mammals (2). Historically, the genus consisted of three taxa: *E. tarda*, *E. hoshinae*, and *E. ictaluri*. Recent investigations into the genetic heterogeneity of *E. tarda*, utilizing comparative phylogenomic analysis, multilocus sequence analysis, DNA-DNA hybridization, and phenotypic characterization, identified the existence of three genetically distinct taxa within the group of organisms traditionally classified as *E. tarda*. This resulted in the adoption of *Edwardsiella piscicida* in 2013 and the recent proposal of *E. anguillarum* sp. nov. in 2015 (3–7).

Based on *gyrB* sequence comparisons, isolate EA181011 was most similar to an *E. piscicida*-like sp. (isolate LADL 05-105) characterized in previous studies (5, 8, 9). A total of 567 Mb of Pacific Biosciences (Pac-Bio) sequences (average length 6 kb) were produced from genomic DNA (gDNA) of EA181011. The longest 40× genome coverage of Pac-Bio reads were error corrected with the remaining Pac-Bio data using the PBcR module within Celera Assembler v.8.2beta (10, 11), then the longest 25× coverage of corrected Pac-Bio sequences were assembled into a single, circular chromosome. In order to correct variations in homopolymer lengths in the consensus Pac-Bio data, we mapped 16× genome coverage of Ion Torrent sequencing reads to the assembled chromosome using Bowtie 2 (12) and produced a consensus sequence using VarScan.v2.3.7 (13).

The circularized and completed genome was submitted to the National Center for Biotechnology Information (NCBI) Prokaryotic Genomes Annotation Pipeline (PGAP) for annotation and submission to GenBank. Furthermore, the genome was submitted for RAST (14, 15) annotation using the Glimmer option.

This *E. piscicida*-like sp. genome consists of one circular chromosome with 3,934,167 bp and 59.1% GC content. PGAP annotation predicted 3,476 genes encoding 3,122 proteins. RNAmmer (16) predicted 8 rRNA operons. Average nucleotide identities (ANI), as calculated by Goris et al. (17), were estimated using an online calculator (http://enve-omics.ce.gatech.edu/ani/) (17). The complete genome of *E. piscicida*-like sp. shares an ANI of 99.75% with *E. tarda* isolate 080813, which was recently proposed as a representative of the novel taxon *E. anguillarum* sp. nov. (4, 7), 94.51% with *E. piscicida* isolate C07-087 (18), 92.48% with *E. ictaluri* isolate 93-146 (19), and 83.86% with *E. tarda* genome FL95-01 (20). Based on comparative RAST analysis with *E. tarda* isolate FL95-01, EA181011 contains 119 unique elements, including components related to mannitol utilization, type I and type VI secretion systems, and a bacteriophage P4 cluster. Isolate EA181011 does not carry any plasmids. The complete, annotated genome of EA181011 will be useful for proper determination of the taxonomic affiliations of future *Edwardsiella* sp. genomes, as well as investigations into this pathogen and its interaction with various hosts.

### Nucleotide sequence accession number.

The complete genome sequence for *Edwardsiella piscicida*-like sp. isolate EA181011 has been deposited in GenBank under the accession number CP011364.
